# Contributions of musculoskeletal rehabilitation in patients after chikungunya fever: a systematic review

**DOI:** 10.1186/s12891-023-06450-6

**Published:** 2023-05-04

**Authors:** Weslley Barbosa Sales, Djavan Gomes Leite, Caroline Sousa Truta Ramalho, Sabrina Gabrielle Gomes Fernandes Macêdo, Gérson Fonseca de Souza, Álvaro Campos Cavalcanti Maciel

**Affiliations:** 1 Graduate Program in Physical Therapy, Federal University of Rio Grande do Norte (UFRN), Avenida Romualdo Galvão 2235, Lagoa Nova, Natal, RN Brazil; 2 Centro Universitário Maurício de Nassau, João Pessoa, Brazil

**Keywords:** Physical therapy modalities, Rehabilitation, Chikungunya fever

## Abstract

**Introduction:**

Chikungunya fever is an infection transmitted by the Chikungunya virus (CHIKV), which is an arbovirus that is transmitted by the mosquitoes Aedes aegypti and Aedes albopictus. The most common sequelae caused by CHIKV are chronic musculoskeletal pain, nerve damage, joint deformation and functional impairment.

**Objective:**

To systematically identify the literature on the contributions of physiotherapy in the treatment of patients with CHIKV sequelae.

**Materials and methods:**

Systematic review of the literature, guided by the recommendations of the Preferred Reporting Items for Systematic reviews and Meta-Analyses (PRISMA). The databases used were PUBMED, LILACS, Scielo and PEDro. Experimental studies and/or full case studies published without language restriction or publication data were included, in which they stood out as contributions of musculoskeletal functional rehabilitation in the treatment of patients with the condition in question. Analytical observational studies, editorial letters, review protocols, reflective studies, literature reviews and articles that do not have an abstract and/or full text available online were excluded.

**Results:**

The search in the databases was carried out between July and August 2022. A total of 4,782 articles were found on the platforms used and 10 articles from the gray literature search. After the duplicate analysis, 2,027 studies were excluded, leaving 2,755 articles that had their titles and abstracts read, of which 600 articles were selected for full reading. After this step, a final sample of 13 articles was eligible for this review.

**Final considerations:**

The most consolidated approaches used in the literature demonstrate that kinesiotherapy, associated or not with electrothermophototherapy, the pilates method and auriculotherapy are useful resources in the treatment of these individuals, significantly inspired by pain relief, improved quality of life and of functionality.

## Introduction

The Chikungunya virus (CHIKV) is a viral disease transmitted by mosquitoes that has become an important public health problem in Brazil in recent years [1]. According to the Brazilian Ministry of Health, between 2014 and 2020, there were more than 1.9 million suspected cases of CHIKV in Brazil, with the highest incidence rates observed in the Northeast and Southeast regions of the country [2]. Rio Grande do Norte (RN), located in the northeast region of Brazil, with a population of around 3.5 million people, had the highest incidence rate, almost six times higher than the national rate (723.1 cases per 100 thousand inhabitants) and 37 deaths caused by the disease [3, 4].

The main symptoms of the acute phase of chikungunya fever occur suddenly with high fever, polyarthralgia and intense myalgia, rash, periarticular edema, fatigue and other symptoms, which appear continuously or inconsistently [1]. Those that affect the joint system, lasting longer than three months is characterized as chronic, being capable of lasting for years or resulting in permanent inflammatory joint disease [2, 5].

In the chronic phase of fever, the functional disability and quality of life of individuals affected by this inflammatory process in the joints can be directly affected [6]. These microlesions, continuously, end up leading to the degradation of bone structures, thus affecting activities of daily living (ADLs), leading to a reduction in their autonomy and Independence [2, 7, 8].

In this sense, the physical rehabilitation process of patients plays a key role in maintaining the functionality of individuals in the chronic phase of the disease, helps to break down of adhesions, influencing the cellular processes of healing, improving lubrication and restoring normal joint mechanics [9]. So, rehabilitation treatment, through a well-structured treatment program, can be useful in the treatment of chronic degenerative joint diseases and/or persistent polyarthralgia [9, 10]. Physiotherapy can be a stand-alone alternative to drugs in the management of joint, tendon and nerve-related pains [10].

Despite being considered an effective treatment in post-Chikungunya fever recovery, there are still few studies that address the effectiveness of physiotherapeutic procedures, mainly systematic reviews, and this study is the first to address this important issue. In addition, this study was carried out due to the scarcity of scientific materials with high methodological quality. Thus, this review will serve as a guide for conduct and approaches for professionals working in the field of musculoskeletal functional rehabilitation. In this sense, this review aimed to systematically identify the literature on the contributions of musculoskeletal rehabilitation in post-chikungunya fever patients.

### Methodology

This study is a systematic review of the literature, following the recommendations of the Preferred Reporting Items for Systematic reviews and Meta-Analyses (PRISMA) [11]. Registration with PROSPERO was carried out, with the approval code: CRD42022363345.

### Identification of the research question

In formulating the research question, the PICO (Population, Intervention, Comparison and Outcomes) strategy, proposed by the Joanna Brigs institute [12]. was used to guide the research problem, thus defining: Population (patients with post-traumatic chikungunya fever); Intervention (musculoskeletal rehabilitation); Comparison (placebo/control) and Outcomes (quality of life, functionality, pain). Thus, the following research question was formulated: “What are the contributions of musculoskeletal rehabilitation in patients with persistent manifestations after CHIKV fever?”. We used “‘’chikungunya fever’’ and functionality as keywords, both were indexed in the Descriptors in Health Sciences/Medical Subject Headings - DeCS/MeSH.

### Identification of relevant studies

This stage was based on meetings with the research team, which defined the planned approach, the eligibility criteria for the studies, the selected databases, as well as the research strategy with their respective descriptors. The eligibility criteria were: experimental studies and/or complete case studies published without language restriction or publication date, in which the contributions of musculoskeletal rehabilitation in the treatment of patients with the condition in question were highlighted.

Observational studies, editorial letters, review protocols, reflection studies, any literature reviews and articles that do not have an abstract and/or full text available online were excluded.

The identification of studies was carried out through an electronic search in the following databases: PUBMED, Latin America and Caribbean Literature in Health Sciences (LILACS), Scielo and on the PEDro platform. The choice of these databases was due to the wide coverage of international and national studies, with public access or available through a library, in addition to the large collection of manuscripts related to the topic.

A review of all reference lists of included studies was also performed to identify additional relevant studies. To also ensure that all relevant information was captured, searches were carried out in a variety of gray literature sources, such as theses and dissertations databases, (inter)national conference proceedings, and in the reference list of selected articles, to identify studies, reports, and conference abstracts relevant to this review.

For gray literature analysis, an extra researcher was invited (JSCD). This researcher was informed about the research objectives and the descriptor used, in order to make an independent search in the gray literature.

### Selection of studies

The selection of studies took place in two stages: in the first, there was a review of titles and abstracts through the Rayyan platform (https://rayyan.qcri.org), eliminating all duplicate articles for this phase [13]. The second stage consisted of reviewing the title and abstract of the eligible articles, and subsequently, the full texts of the studies selected in the second stage were read. For both stages, the process was carried out by two researchers (WBS and DGL), who were instructed on the eligibility criteria and were responsible for reading and extracting the data, while a third evaluator (JSCD) was responsible for solving problems related to possible doubts and/or divergences in the selection.

### Data extraction

Data were extracted using a table developed by the research team, which aims to collect information such as: authors, year, place of publication, outcomes, intervention and expected effects. This instrument was previously tested by all reviewers before its final application, to ensure that the information captured was accurate.

Data collection was performed in pairs, where two reviewers (WBS and DGL) independently extracted data from all included studies. In the end, the study collections of each reviewer were compared to visualize possible discrepancies and, if any, they were reviewed and analyzed by the research group, thus ensuring consistency between reviewers.

### Methodological quality

Study quality was assessed using the Joanna Briggs Institute (JBI) assessment tools and recommendations. This assessment aimed to analyze the methodological quality of the studies and determine the extent to which a study addressed the possibility of bias in its design, conduct and analysis [14].

Each study was categorized according to the percentage of positive responses. The risk of bias was considered high when the study obtained up to 49% of answers classified as yes, moderate when it obtained from 50 to 69% of positive responses and low when the study reached more than 70% of the score [11–14]. The process was conducted by two independent reviewers (WBS and DGL). Disagreements were resolved by another reviewer (JSCD).

### Ethical issues and patient and public engagement

No patients were involved in this research. Furthermore, this review did not require ethical approval, as it consisted of analyzing and collecting information from publicly available documents.

## Results

**Fig. 1:**
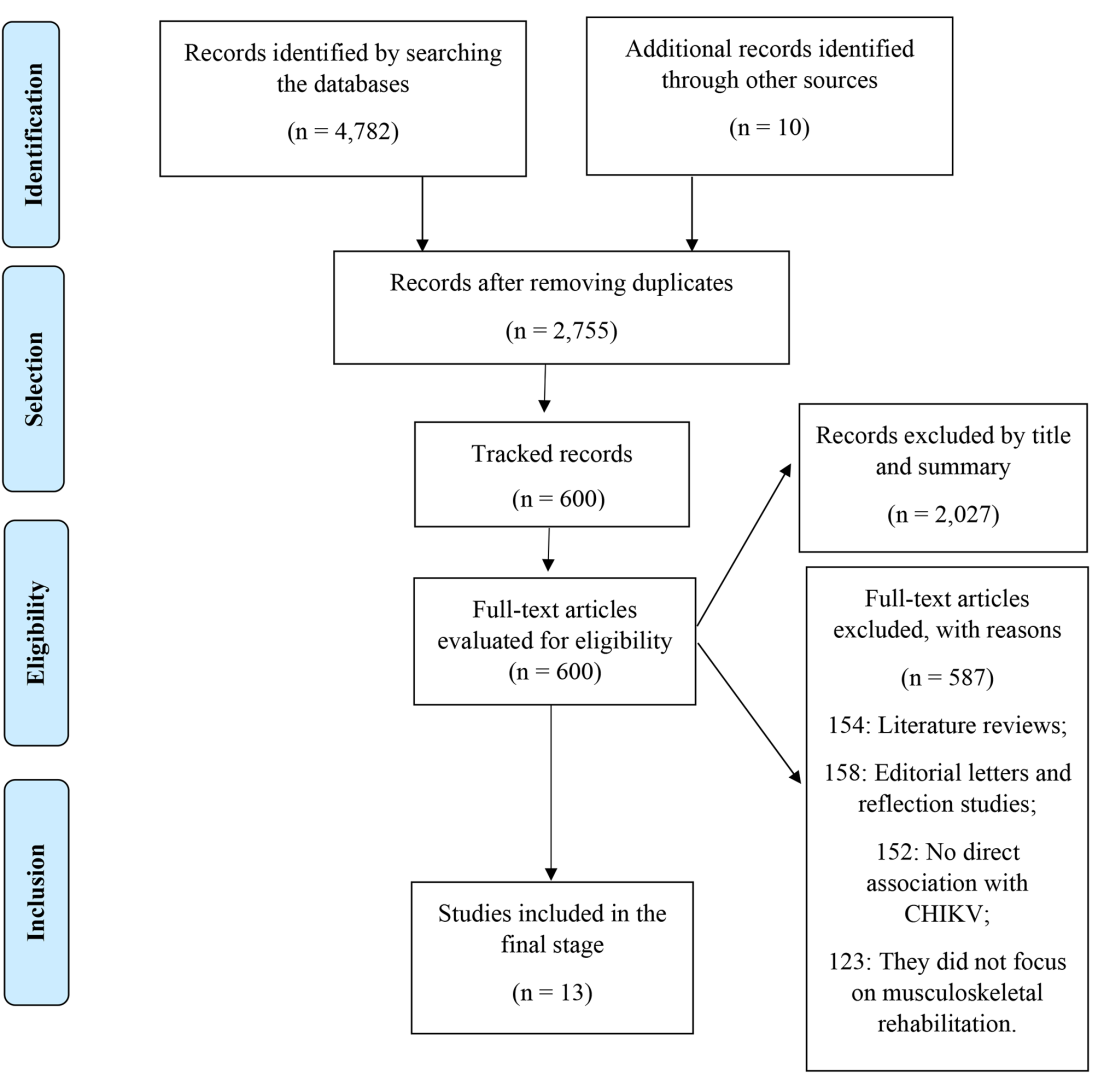
Prism flowchart for systematic reviews

The database search was carried out between July and August 2022. A total of 4,782 articles were found on the platforms used and 10 articles from the gray literature search carried out in the CAPES Catalog of Theses and Dissertations After the duplicate analysis, 2,027 studies were excluded, leaving 2,755 articles that had their titles and abstracts read, of these, 600 articles were selected for reading in full. After this step, a final sample of 13 articles was eligible for this review. More details can be found in Fig. 1. While, the characteristics of the included studies are shown in Table 1.

**Table 1:** Details of the studies included according author, year, population, country, study designer and duration of sequels

Author (year)	Population /sample	Country of origin	Study designer	Sequel time
Ribeiro et al., 2016 [15]	1 male (57 years old)	Brazil	Case report	5 months after initial symptoms) and 10 days later.
Oliveira and Silva [9]	1 woman (35 years old)	Brazil	Case report	After 5 months of the onset of symptoms, in July 2016.
Silva-Filho et al., 2018 [16]	20 individuals (both sexes between 18 and 65 years old)	Brazil	Randomized controlled trial	After 6 months of the onset of symptoms.
Coutinho et al., 2018 [17]	102 individuals (both sexes over 18 years old)	Brazil	Retrospective, analytical study	From 0 to 90 days after symptoms
Oliveira, et al., 2019 [18]	51 individuals (both sexes over 18 years old)	Brazil	Randomized controlled trial	More than 3 months
Neumann et al., 2019 [19]	31 subjects (both sexes aged 18–75 years)	Brazil	Randomized controlled trial	More than 3 months
Siqueira, et al., 2019 [20]	35 individuals (both sexes over 18 years old)	Brazil	Controlled and randomized clinical trial	More than 3 months
Neumann et al., 2019 [21]	31 subjects (both sexes aged 18–75 years)	Brazil	Controlled and randomized clinical trial	More than 3 months
Oliveira [22]	51 individuals (both sexes over 18 years old)	Brazil	Randomized controlled trial	More than 3 months
Silva et al., 2020 [23]	21 women (40–60 years old)	Brazil	Randomized controlled trial	Does not report
Tenório et al., 2020 [24]	2 women (47–58 years old)	Brazil	Case reports	Does not report
Souza et al., 2021 [25]	58 women (28 to 70 years old)	Brazil	Randomized clinical trial.	More than 3 months
Almeida et al. 2021 [26]	21 individuals (both sexes over 18 years old)	Brazil	Quasi-experimental, uncontrolled and non-randomized study, with pre- and post-test in a single group	After the acute phase of infection, which is considered to be up to 10 days

### Characteristics of the studies

A total of 391 individuals composed the sum of the samples of the included studies, the smallest comprising 1 participant, while the largest sample analyzed 102 patients. Regarding gender, most articles used participants of both sexes (84.7%), except for 2 studies, which were composed only of women and 1 composed only of men (Table 1). Of the 13 articles included, all were published after 2016, carried out in Brazil, and the most used study design was the randomized controlled trial (61.52%) (Table 1).

### Time of sequels

More than half of the articles included (53.9%) had participants with post-Chikungunya fever sequels equal to or longer than 3 months.

### Outcomes

With regard to outcomes, it was observed that pain intensity was the most explored variable in the literature (92.4%), followed by quality of life (69.3%) and range of motion (38.5%) (Table 2). The perception and change in health status and satisfaction with the treatment used were explored in only two studies (15.4%) [19, 21]. Other variables such as edema (7 0.7%), muscle strength (15.4%), and blood factors (7.7%) [9, 23, 26] were the least observed outcomes.

**Table 2:** Details of the included studies according to author, year, participants, outcomes and instruments, intervention and effects found

Author (year)	Participants	Outcomes and instruments	Intervention	Effects found (From according to the numbering of outcomes)
Ribeiro et al., 2016 [15]	Case report (n = 1)	1) Pain intensity (VAS); 2) Quality of life (SF-36).	Duration: ten sessions. Protocol: ultrasound continuous with a frequency of 1 MHz, Infrared Laser with a dosage of 4 J and 3s per dot; TENS-burst with a pulse width of 250 us and a frequency of 2 Hz.	1) Reduction of pain measured using the VAS instrument; 2) Improved quality of life measured using the SF-36 instrument.
Oliveira and Silva [9]	Case report (n = 1)	1) Pain intensity (VAS); 2) Range of motion (goniometer); 3) Muscle strength (TEM); 4) Edema (perimetry); 5) Capacity functional (TC10), Quick dash12 and Lequesne Scales version in Portuguese, for the upper and lower limbs, respectively.	Duration: four weeks, one turn per week. Protocol: Kinesiotherapy classical and manual therapy.	1) Reduction of pain measured using the VAS instrument; 2) There was no improvement in range of motion measured by goniometry; 3) There was no improvement in muscle strength measured by the TEM instrument; 4) No improvement was observed in edema measured using the perimetry instrument; 5) There was an improvement in the functional capacity measured using the TC10 instrument.
Silva-Filho et al., 2018 [16]	tDCS group (n = 10) sham -tDCS group (n = 10)	1) Pain intensity (VAS; BPI); 2) Quality of life (SF-36)	Duration: Five days consecutive; Protocol: stimulation current transcranial _ direct (tDCS) with current 2 mA constant for 20 min (30 s ramp-down); Sham - tDCS with current 2 mA constant was delivered only for 30 s (10 s ramp-up) of the 20 min	1) Reduction of pain measured using the VAS and BPI instruments; 2) Improved quality of life measured using the SF-36 instrument.
Coutinho et al., 2018 [17]	Group in phase acute / subacute (n = 59) Group in phase chronic (n = 43)	1) Pain Intensity (NPRS); 2) assessment of mobility and balance (TUG); 3) Functional capacity (SPPB).	Duration: Five weeks; Protocol: auriculotherapy in points specific acupuncture and _ auriculotherapy sham em points no specific.	1) Pain reduction was not observed using the NPRS instrument; 2) Improvement in balance and mobility measured using the TUG instrument; 3)Improvement of functional capacity through the SPPB instrument.
Oliveira, et al., 2019 [18]	Intervention group Pilates method (n = 22) Control group (n = 20)	1) Pain intensity (VAS); 2) Quality of life (SF-36); 3) Range of motion (goniometry and flexibility test with wells bank).	Duration: 24 sessions, twice per week; Protocol: Pilates method one series of 6 to 12 repetitions; the training he was Divided in training A (22 exercises) and training B (18 exercises ).	1) Reduction of pain observed through the VAS instrument; 2) Improvement in quality of life observed through the SF-36 instrument; 3) Improvement in range of motion observed through goniometry and flexibility test.
Neumann et al., 2019 [19]	Exercise resisted (GER) (n = 15) Control (GC) (n = 16)	1) Pain intensity (VAS); 2) Quality of life (SF-36); 3) Functional capacity (40mWT); (TSL30s); (TSD4d); (DASH); 4) perception of treatment (PGIC).	Duration: 24 sessions twice per week; Protocol: Resistance exercises _ elastic with intensity load moderate and progressive, evaluated through testing a repetition maximum.	1) Reduction of pain observed through the VAS instrument; 2) Improvement in quality of life observed through the SF-36 instrument; 3) Improvement of functional capacity observed through the instruments 40mWT, TSL30s, TSD4d, DASH; 4) Improvement of the perception of the patient’s treatment through the PGIC instrument.
Siqueira, et al., 2019 [20]	They were accompanied (n = 35)	1) Pain intensity (VAS);	Duration: Six sessions; Protocol: Auriculotherapy with mustard seeds and tape adhesive in points with effect painkiller.	1) Reduction of pain observed through the VAS instrument;
Neumann et al., 2019 [21]	Exercise resisted (GER) (n = 15) and Control (GC) (n = 16)	1) Pain intensity (VAS); 2) Quality of life (SF-36); 3) Functional capacity (TC40m; TSL30s; TSD4d; DASH); 4) perception of treatment (PGIC).	Duration: 24 sessions to the over 12 weeks; Protocol: exercise with resistance elastic with intensity load moderate and progressive, evaluated through testing a repetition maximum	1) Reduction of pain observed through the VAS instrument; 2) Improvement in quality of life observed through the SF-36 instrument; 3) Improvement of functional capacity observed through the instruments 40mWT, TSL30s, TSD4d, DASH; 4) Improvement of the perception of the patient’s treatment through the PGIC instrument.
Oliveira [22]	Pilates group (n = 26) one group control (n = 25)	1) Pain intensity (VAS); 2) Quality of life (SF-12); 3) Range of motion (Goniometry) 4) Capacity functional (HAQ)	Duration: 24 sessions twice per week; Protocol: Mat Pilates Method 50 min.	1) Reduction of pain observed through the VAS instrument; 2) Improvement in quality of life observed through the SF-12 instrument; 3) Improvement in range of motion observed through goniometry; 4) Improvement of functional capacity observed through the HAQ instrument.
Silva et al., 2020 [23]	They were selected (n = 21)	(1) Peroxidation lipid; (2) Protein oxidation; 3) Antioxidant system no enzymatic	Duration: 12 training sessions, lasting 40 min; Protocol: Exercise physicists with intensity of 50–70% of the frequency cardiac maximum	1) Improvement of lipid peroxidation processes observed through laboratory tests; 2) Improvement in protein oxidation observed through laboratory testing; 3) There was improvement in the non-enzymatic antioxidant system observed through laboratory tests.
Tenório et al., 2020 [24]	Case reports (n = 2)	1) Pain intensity (VAS); 2) Quality of life (SF-36)	Duration: 14 sessions; Protocol: Electrothermotherapy, kinesiotherapy and manual therapy	1) Reduction of pain observed through the VAS instrument; 2) Improvement in quality of life observed through the SF-36 instrument.
Souza et al., 2021 [25]	Activate-tDCS (n = 29) sham-t DCS (n = 30)	1) Pain intensity (VAS); 2) Capacity functional (HAQ)	Duration: 6 sessions, two weeks; Protocol: stimulation with an anode placed at M1 region and the cathode over the supraorbital region	1) Reduction of pain observed through the VAS instrument; 2) Improvement of functional capacity observed through the HAQ instrument.
Almeida et al. 2021 [26]	Sample composed by (n = 21)	1) Pain intensity (VAS); 2) Quality of life (SF-36); 3) Range of motion and ability functional (Goniometry); 4) strength of grip (hand dynamometry)	Duration: ten sessions; Protocol: mobilizations joints, stretching, exercises Resumen aerobic, active-resisted, active - free and resource electrothermotherapeutics	1) Reduction of pain observed through the VAS instrument; 2) Improvement in quality of life observed through the SF-36 instrument. 3) Improvement in range of motion observed through goniometry and flexibility test; 4) Increase in grip strength observed using a hand dynamometry.

Note: VAS - Visual analogue scale; SF-36 - Medical Outcomes Study 36 - Item Short-Form Health Survey; HAQ - Health Assessment Questionnaire; TEM - sphygmomanometer test modified; 40mWT − 40-meter walk test; SF-12 - Short-Form Health Survey; TC10–10 m walk test; TSL30s - One-way sitting and standing test chair; TSD4d - up-down test ladder; DASH - Disabilities of the Arm, Shoulder, Hand; PGIC - Patient Global Impression of Change Scale; SPPB - Test the Short Physical Performance Battery; BPI - McGill Pain Questionnaire and Brief Pain Inventory; NPRS - scale Pain Numeracy; TUG - Timed Up and Go;

### Intervention

With regard to duration and frequency, it was observed that most of the studies (92.4%) of the sample carried out interventions over five sessions, twice a week. However, only one study [9] had a shorter duration, once a week for four weeks. With regard to the protocols, it was shown that kinesiotherapy associated or not with electrothermophototherapy was the most used therapeutic approach (69.3%), followed by the pilates method and auriculotherapy (15.4% each) [20–22].

### Effects found

Among the effects found, with regard to people who underwent musculoskeletal rehabilitation, the variable pain intensity was the most expressive in the analyzed literature (84.7%), measured using the visual analogue scale (VAS), McGill Pain Questionnaire and Brief Pain Inventory [16, 17] and the numeric pain scale [18], followed by the quality of life variables (53.9%) measured using the Short Form Health – 36 (SF -36) [19, 21] and 12-item health survey (SF-12) [9]; mobility and balance measured through the timed up and go (TUG) [17, 19, 26] and functional capacity measured by the Health Assessment Questionnaire (HAQ) and short physical performance battery (SPPB) [18]; perception/change in health status and satisfaction, measured by the Patient Global Impression of Change Scale (PGIC) (15.4%) [19, 23, 26]; and range of motion (23.1%), measured using goniometry [17, 20, 22].

### Methodological quality

When evaluating the methodological quality and the risk of bias of the selected references, according to the study design, it is observed that most articles (53,83%) have a low risk of bias, with an average of 72.7% of positive responses. Meanwhile, 5 studies (38,45%) were classified as having a moderate risk of bias, with a range between 54.5% and 63.6% [9, 16, 20, 23, 26]. Only one study (7,69%) was classified as having a high risk of bias of 45.4% [24]. Detailed evaluations of each article can be seen in Tables 3 and 4 respectively.

**Table 3:** Degree of quality of evidence of the included studies

Authors and year	Corresponding criteria and scores												
	#1	#2	#3	#4	#5	#6	#7	#8	#9	#10	#11	Total	%
Ribeiro et al., 2016 [15]	NA	NA	1	1	0	1	1	1	1	NA	NA	6	54,5
Oliveira and Silva 2017 [9]	NA	NA	1	1	0	1	1	1	1	NA	NA	6	54,5
Silva-Filho et al., 2018 [16]	1	1	1	0	NA	1	1	1	1	NA	1	8	72,7
Coutinho 2018 [17]	1	1	1	0	NA	1	1	1	1	NA	1	8	72,7
Oliveira 2018 [18]	1	1	1	0	NA	1	1	1	1	NA	1	8	72,7
Neumann et al., 2019 [19]	1	1	1	0	NA	1	1	1	1	NA	1	8	72,7
Siqueira, et al., 2019 [20]	NA	1	1	0	NA	1	1	1	1	NA	1	7	63,6
Neumann et al., 2019 [21]	1	1	1	0	NA	1	1	1	1	NA	1	8	72,7
Oliveira et al., 2019 [22]	1	1	1	0	NA	1	1	1	1	NA	1	8	72,7
Silva et al., 2020 [23]	NA	1	1	0	NA	1	1	1	1	NA	1	7	63,6
Tenório et al., 2020 [24]	NA	NA	1	0	NA	1	1	1	1	NA	NA	5	45,4
Souza et al., 2021 [25]	1	1	1	0	NA	1	1	1	1	NA	1	8	72,7
Almeida et al., 2021 [26]	NA	1	1	0	NA	1	1	1	1	NA	1	7	63,6

Note: 0 = No; 1 = Yes; NA = Not applicable; #1: Were the two groups similar and recruited from the same population? #2: Were exposures measured similarly to assign people to exposed and unexposed groups? #3: Was the exposure measured validly and reliably? #4: Were confounding factors identified? #5: Have trategies for dealing with confounders been stated?; #6: Were the groups/participants free of the outcome at baseline (or at the time of exposure)?; #7: Were the results measured validly and reliably?; #8: Was the follow-up time reported long enough for results to occur?; #9: Was follow-up complete and, if not, were reasons for loss to follow-up described and explored?; #10: Were strategies used to deal with incomplete follow-up?; #11: Was the appropriate statistical analysis used?

**Table 4:** Degree of methodological quality and risk of bias

Authors (year)	Methodological quality (%)	Risk of bias
Ribeiro et al., 2016 [15]	45,4	High (Up to 49%)
Oliveira and Silva 2017 [9]	54,5	Moderate (50 the 69%)
Silva-Filho et al., 2018 [16]	54,5	Moderate (50 the 69%)
Coutinho 2018 [17]	63,6	Moderate (50 the 69%)
Oliveira 2020 [18]	63,6	Moderate (50 the 69%)
Neumann et al., 2019 [19]	63,6	Moderate (50 the 69%)
Siqueira et al., 2019 [20]	72,7	Low (above 70%)
Neumann et al., 2019 [21]	72,7	Low (above 70%)
Oliveira et al., 2019 [22]	72,7	Low (above 70%)
Silva et al., [23]	72,7	Low (above 70%)
Tenório et al., 2020 [24]	72,7	Low (above 70%)
Souza et al., 2021 [25]	72,7	Low (above 70%)
Almeida et al., 2021 [26]	72,7	Low (above 70%)

## Discussion

This systematic review aimed to systematically identify the literature on the contributions of musculoskeletal rehabilitation in the treatment of patients with CHIKV sequelae. A scarcity of intervention studies was observed in patients with post-Chikungunya fever sequels, despite the great importance of the topic. This scarcity may be related to the great challenges of working with this population, such as the lack of well-established intervention protocols, ethical issues and high frequencies of chronic sequels and dropout, making it difficult to carry out these studies.

Ribeiro et al. [15] clarifies that the application of low-intensity laser and ultrasound are important in the treatment of chronic rheumatological pathologies, showing that these resources have the objective of reducing inflammation, pain and joint stiffness, seeming to be a good intervention for patients with post-chikungunya fever sequels.

Corroborating with Ribeiro et al. [15], the study by Oliveira and Silva [9] clarifies that persistent polyarthralgia after chikungunya fever is a common clinical manifestation in cases of CHIKV infection. In this scenario, Oliveira and Silva [9] carried out a musculoskeletal rehabilitation program based purely on kinesiotherapy (therapeutic exercises and manual therapy) for four weeks. The results of this study indicated that kinesiotherapy as an isolated treatment was effective in increasing muscle strength, range of motion, decreasing edema and improving functional capacity, in addition to notably contributing to the reduction of pain.

In addition to kinesiotherapy, other studies demonstrate the benefits of the Pilates method in this context, reducing pain in patients with chronic musculoskeletal manifestations after Chikungunya fever. An example of this is the study carried out by Oliveira [18], where two intervention groups were compared (Pilates and the standard treatment), and it was seen that individuals who did Pilates method had a significant improvement in pain and quality of life [21].

The literature also demonstrates that resistance exercise has satisfactory effects in the treatment of rheumatological diseases, and may be a non-pharmacological strategy in the treatment of sequelae caused by CHIKV. In this sense, the study by Neumann [19] carried out a study to develop a protocol of progressive resistance exercises for the treatment of individuals with chronic musculoskeletal manifestations of chikungunya and to evaluate its effectiveness in improving functionality, pain and quality of life. And as a result, the resistance group obtained benefits in improving functionality and reducing pain in patients with chronic musculoskeletal manifestations of chikungunya when compared to control group.

Complementing the evidence above, the study by Silva et al. [23] sought to evaluate the effect of physical exercise on the oxidative balance of lymphomononuclear cells (PBMCs) in patients with chronic infection by the CHIKV. It is observed that oxidative stress can cause damage to cartilage, the main protective tissue of bones, while chronic inflammation causes a series of gradual changes in the immune system. That said, the study showed that a physical exercise protocol, with twelve training sessions, lasting forty minutes and intensity of 50–70% of the maximum heart rate did not minimize oxidative stress in PBMCs of patients with chronic infection by chikungunya after the physical exercise protocol used in this study. The authors suggest new clinical studies in order to consolidate and complement this evidence [23].

Bringing an innovative proposal, the study by Silva-filho et al., [16] sought to investigate the hypothesis that Transcranial Direct Current Stimulation (tDCS) would improve pain and functionality in individuals with chronic arthralgia after Chikungunya fever. In this study, twenty patients in the chronic phase of chikungunya were randomized into two groups, one receiving active tDCS and the other sham tDCS (control). The results of this research showed that tDCS reduces pain levels with clinically significant changes for patients with Chikungunya.

In the scenario of integrative and complementary health practices, the study by Coutinho [17] sought to evaluate the effectiveness of auriculotherapy, complementary to standard drug treatment, in improving pain and mobility of symptomatic individuals after Chikungunya Fever, as well as the predictive factors for disability at different stages of the disease.

Fifty individuals diagnosed with post-Chikungunya fever sequelae were divided into two intervention groups: auriculotherapy at specific acupuncture points and sham auriculotherapy at non-specific points. Both groups received five treatment sessions performed once a week over five weeks. The results demonstrate that auriculotherapy is effective in improving mobility limitation after Chikungunya fever, and that individuals in the acute/subacute phase of the disease have greater disability, with mobility limitation being the main predictor of functional decline.

Corroborating with the evidence from the study by Coutinho [17], the study by Siqueira et al. [20], sought to evaluate the effectiveness of auriculotherapy in the control of chronic pain caused after CHIKV infection. Thirty-five individuals with chronic joint pain were followed during six sessions of auriculotherapy and the results of this study not only demonstrate the efficiency of this technique, but also clarify that the technique can be an adjuvant resource in the treatment of chronic joint pain in these patients, not just as a complementary treatment [20].

Corroborating the above, Tenório et al. [24] clarifies that fourteen physical therapy sessions with joint mobilizations, motor coordination training, analgesia and active and passive stretching and muscle strengthening exercises are important approaches in the treatment of post-Chikungunya fever patients. In their study, the results demonstrate that physiotherapeutic follow-up provided a decrease in pain intensity and an increase in the quality of life of patients with arthralgia after infection by arboviruses.

Reinforcing the results of Tenório et al. [24], the study by Almeida et al. [26] aimed to verify the influence of a multimodal physiotherapeutic approach on pain, quality of life, mobility and handgrip strength in patients with Chikungunya virus sequels. The sample consisted of twenty-one participants with persistent arthralgia after CHIKV infection.

The approach consisted of joint mobilizations, stretching, aerobic, active-resistance, active-free exercises and electrothermotherapeutic resources, totaling ten sessions. The results of this research indicate that a physiotherapeutic approach with electrothermotherapeutic and kinesiotherapeutic resources reduces pain, increases mobility, quality of life and handgrip strength in individuals with CHIKV sequels.

### Strengths and limitations of the study

This study has some important limitations. The first concerns the variation in the age range of the participants in the included studies, ranging from 18 to 75 years. As there is a scarcity of experimental/intervention studies, it was not possible to develop a subgroup analysis in this systematic review, so information on specific groups (adults and elderly) could not be compared. The second limitation refers to the assessment of the risk of bias of the included studies, as almost half of the sample ranged from 45.4 to 63.3 (high and moderate risk of bias, respectively).

As for strengths, this is the first review that aims to carry out a survey on the contributions of musculoskeletal rehabilitation in the treatment of patients with sequelae after Chikungunya fever, in addition to specifically each type of intervention, outcomes and effects found.

### Final considerations

The results of this study show the contributions of musculoskeletal functional rehabilitation in the treatment of patients with CHIKV sequelae. The most consolidated approaches in the literature used demonstrate that kinesiotherapy, associated or not with electrothermophototherapy, the pilates method and auriculotherapy, are useful resources in the treatment of these individuals, contributing significantly to pain relief, improved quality of life and functionality.

In this way, this study encourages the production of new research on a similar topic, in order to further emphasize the importance of the rehabilitative mechanisms that physiotherapy and other related professions use in patients with musculoskeletal manifestations after Chikungunya fever. Another point that needs greater exploratory effort is the early and accurate diagnosis of Chikungunya, since the signs and symptoms can be confused with other pathologies, such as rheumatoid arthritis, which can be considered a confusing fact in the treatment of sequels patients.

## Data Availability

The datasets used and/or analyzed during the current study are available from the corresponding author on reasonable request.
